# Fully automated coronary artery calcium score and risk categorization from chest CT using deep learning and multiorgan segmentation: A validation study from National Lung Screening Trial (NLST)

**DOI:** 10.1016/j.ijcha.2024.101593

**Published:** 2025-01-02

**Authors:** Sudhir Rathore, Ashish Gautam, Prashant Raghav, Vijay Subramaniam, Vikash Gupta, Maanya Rathore, Ananmay Rathore, Samir Rathore, Srikanth Iyengar

**Affiliations:** a Department of Cardiology, Frimley Park Hospital NHS Foundation Trust, Camberley, Surrey, UK; b Honorary Reader, University of Surrey, Guildford, UK; c KardioLabs AI, Jacksonville, FL, USA; d Research Associate, University of Waterloo, Waterloo, Ontario, Canada; e Department of Radiology, Mayo Clinic, Jacksonville, FL, USA; f Department of Radiology, Frimley Park Hospital NHS Foundation Trust, Camberley, Surrey, UK; g Medical Student, Newcastle University, Newcastle, UK; h Medical Student, University of Bristol, Bristol, UK

**Keywords:** Coronary artery calcium score, Artificial Intelligence, Chest CT, Coronary artery disease, Risk categorization

## Abstract

**Background:**

The National Lung Screening Trial (NLST) has shown that screening with low dose CT in high-risk population was associated with reduction in lung cancer mortality. These patients are also at high risk of coronary artery disease, and we used deep learning model to automatically detect, quantify and perform risk categorisation of coronary artery calcification score (CACS) from non-ECG gated Chest CT scans.

**Materials and methods:**

Automated calcium quantification was performed using a neural network based on Mask regions with convolutional neural networks (R-CNN) for multiorgan segmentation. Manual evaluation of calcium was carried out using proprietary software. This study used 80 patients to train the segmentation model and randomly selected 1442 patients were used for the validation of the algorithm. We compared the model generated results with Ground Truth.

**Results:**

Automatic cardiac and aortic segmentation model worked well (Mean Dice score: 0.91). Cohen’s kappa coefficient between the reference actual and the interclass computed predictive categories on the test set is 0.72 (95 % CI: 0.61–0.83). Our method correctly classifies the risk group in 78.8 % of the cases and classifies the subjects in the same group. F-score is measured as 0.78; 0.71; 0.81; 0.82; 0.92 in calcium score categories 0(CS:0), I (1–99), II (100–400), III (400–1000), IV (>1000), respectively. 79 % of the predictive scores lie in the same categories, 20 % of the predictive scores are one category up or down, and only 1.2 % patients were more than one category off. For the presence/absence of coronary artery calcifications, our deep learning model achieved a sensitivity of 90 % and a specificity of 94 %.

**Conclusion:**

Fully automated model shows good correlation compared with reference standards. Automating the process could improve diagnostic ability, risk categorization, facilitate primary prevention intervention, improve morbidity and mortality, and decrease healthcare costs.

## 1 Introduction

Cardiovascular disease (CVD) is the common cause of death and 50% of the CVD related deaths have no previous cardiac symptoms or diagnosis, therefore, risk stratification in asymptomatic individuals is paramount [1]. Several clinical tools for risk categorization and risk factor analysis are used to risk stratify the high-risk individuals and initiate the preventative therapy [[2], [3], [4]]. Coronary artery calcification (CAC) has shown and proved to be important determinant of cardiovascular risk stratification and marker of coronary artery disease (CAD) [[2], [5]]. The National Lung Screening Trial (NLST) was executed to evaluate whether annual screening with low dose computed tomography (LDCT) scans could reduce mortality from lung cancer in high-risk individuals [6]. There is an opportunity to identify and detect CAC form non contrast chest CT and risk stratify the patients of having CAD. Studies have shown CAC could be visually identified and quantified from non-contrast chest CT, into mild, moderate, and or severe and could be specified to the coronary artery distribution [[4], [7], [8]].

There has been agreement from international societies for routine assessment, reporting, and quantification of the CAC from the ungated non contrast chest CT [[4], [9]]. However, CAC reporting and quantification from non-contrast chest CT is time consuming and require extra training and resources. Currents standard practice across the board relies primarily on visual evaluation of the CT scan and then subjectively categorise the degree of CAC. This practice is variable due to vast interobserver reliability [[10], [11], [12]] and limited time for scan [13]. Many times, this important information is missed due to focus on the primary indication of the study. Artificial Intelligence (AI) has experienced an exponential growth over the last few years. This is due to the widespread application of machine learning (ML), particularly deep learning (DL), that has led to the development of highly accurate models that bridge the gap of limited time and interobserver variability [14]. Previous studies have shown using AI and ML to identify CAC on the non-contrast chest CT with variable sensitivity and specificity and some false positive results, and not much data available for risk categorization and different calcium scores [[16], [15], [17], [18], [19], [20], [21]]). Therefore, we have developed an in-house multi-segmentation model to reduce the false positive rates for automated coronary artery calcium detection, quantification, and risk categorization.

## 2 Material and methods

### 2.1 Data acquisition

The study was submitted to review by the NIH NCI (National Institute of Health supported National Cancer institute), Cancer Data Access System and was approved as NLST-774, on March 30, 2021.Data from this cohort has not been published before. This is a retrospective review of Imaging Data acquired as below. Goal of this study is to validate the accuracy AI algorithm to detect and quantify coronary artery calcification from Chest CT scans.

Data from 15,000 participants of the NLST trial were acquired. The NLST was approved by the institutional review board at each of the participating institutions in United States of America (U.S.A) and informed consent was obtained from all participants. Image review for this study was compliant with the Health Insurance Portability and Accountability Act. Eligibility criteria were patient age 55–74 years and 30 pack-years or more of cigarette history. We have used data from randomly identified 1442 patients from this group to validate our algorithm. NLST database has been previously used by other investigators to test different algorithms to detect and quantify coronary artery calcium [[18], [19]].

### 2.2 Study population and participants

#### 2.2.1 Imaging protocol

Low-dose CT imaging acquisition parameters were as follows: 120 kVp; 40–80 mAs (depending on body habitus); detector collimation, 1.25–2.5 mm; reconstruction interval, 1.0–2.5 mm; and soft-tissue reconstruction algorithm [22]. All the Images used were unenhanced and non −ECG gated.

### 2.3 Ground truth (Real true Calcium) assessment

#### 2.3.1 CAC identification and segregation

A Cardiologist with Level 2 CT accreditation (S.R., with 10 years of experience in cardiovascular CT image and reported >5000 studies) manually performed CAC identification and segregation on an axial and sagittal view of the CT images. CAC was identified using 130 Hu as a threshold in the coronary artery distribution and isolated. [23].

#### 2.3.2 CAC calculation and estimation

Agatston score (AS), was calculated based on manual identification and segregation of the coronary calcification and outlining. All the identified calcium was then combined and calculated using proprietary calcium scoring software as per clinical calcium scoring standards [16]. The result of CACS was respectively recorded at total and vessel-specific levels, i.e., left main coronary artery (LMCA), left anterior descending coronary artery (LAD), left circumflex coronary artery (LCX) and right coronary artery (RCA).

#### 2.3.3 Risk categorization

Coronary artery calcium score (CACS) was categorized as per Coronary Artery Calcium Data and Reporting (CAC-DRS) recommendation, [24]. CACS were recorded as 5 grades: Category 0 (AS 0), Category I (AS 1-100), Category II (AS 101-400), Category III (AS 401-1000), Category IV (AS >1000).

### 2.4 AI model detected CAC and risk categorization.

CT scans of the patients included in the study were passed through the AI model as described below and CAC was identified and quantified. Subsequently total CAC in the coronary arteries area was calculated and risk categorization was performed. This process was supervised and arbitrated by the trained Cardiologist.The Experimentation is as following:

### 2.5 Deep learning model

**Fig. 1:**
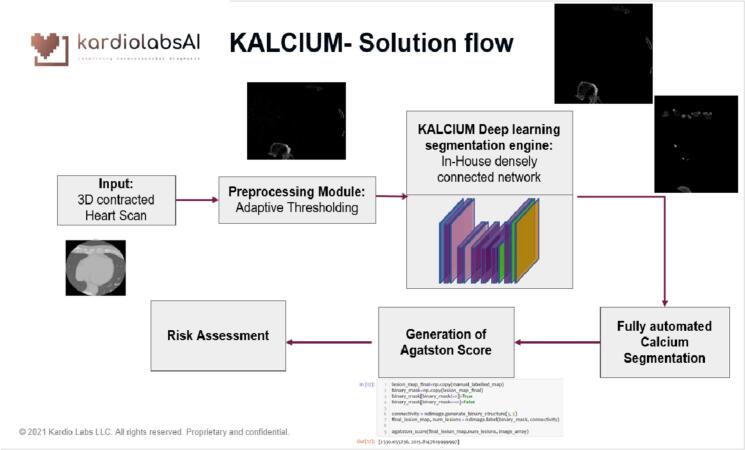
AI Solution Overview.

We have developed a novel approach for calcium detection, classification, and quantification in a totally automatic fashion from the given CT volume. Main steps in building this automated solution are namely (Fig. 1):

1. Multi Organ Segmentation: Cardiac, Aorta, and epicardial Fat segmentation.
2. Coronary artery region segmentation.
3. True calcium segmentation.
4. Calcium Scoring.

The input cardiac CTs are uploaded using a software as a service (SAS) based platform developed by our team. The data was anonymized to remove patient health information (PHI) information from the scans. The models were trained on eight NVIDIA Tesla V100 Graphic Processing Units (GPUs) running on Amazon Web Services (AWS) cloud infrastructure (Fig. 1). The input to the model is a 3D non-contrast chest CT scan and images are consistent with 512 × 512 format. The images were resized to match the input size. This is followed by morphological dilation and anisotropic smoothing. Next, we segment the calcium in the heart by first using intensity and volume threshold to filter calcium in the non-contrast CT and then removing noise as well as non-useful calcium in the bone and extra cardiac calcium. The AS for each artery region is then computed as the sum of ASs of calcium group in that artery region across all slices.

### 2.6 Pre-processing

Non contrast Chest CT examinations vary widely in resolution, field of view, noise characteristics and construction kernels. Therefore, additional pre-processing is required before application of the CAC identification using dedicated software and the automated model. We have performed following pre-processing steps:

Pre-processing using **Image re-sizing** to standard the size.

**Morphological Dilation**: This is a mathematical operation that adds pixels to the boundaries of objects in an image. This makes objects more visible and fill in the small holes in an image. Dilation uses structuring element to probe and expand the shapes in an image. The number of pixels added depends on the size and shape of the structuring element. This is commonly used in image procession and computer vision.

**Anisotropic smoothing**: This aims at reducing image noise without removing parts of the image content, typically edges, lines or other details that are important for the interpretation of the image. This results pixel intensity values to diffuse over neighbouring pixels, with the diffusion at a point being inversely proportional to the local contrast. This enhances the edges and generate plausible detail in images.

We also performed re-alignment of the images so that all images are of the same size and constitution.

### 2.7 Multi organ segmentation: cardiac, aortic, and epicardial fat segmentation

**Fig. 2:**
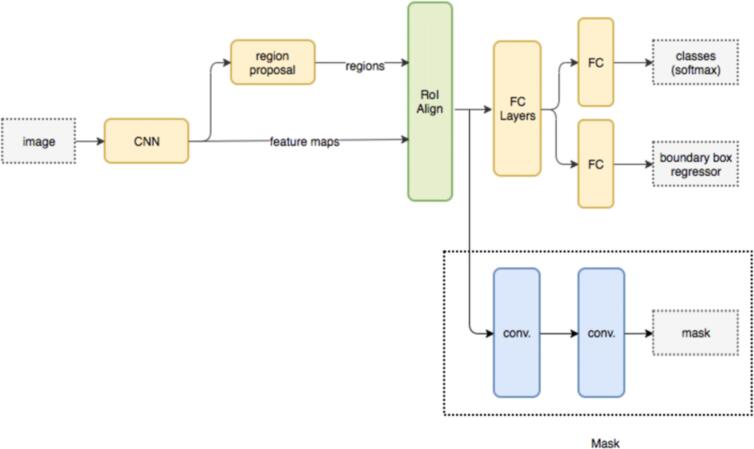
Outline of the Deep Network framework Kardionet. CNN: Convolutional Neural Network; FC: Fully Connected; ROI: Region of Interest.

We used our in-house modified connected deep network framework KardioNet, which has been customized to multi organ segmentation of the cardiac scan. The proposed method is based on the well-known multi-instance segmentation method, Mask R-CNN. The architecture consists of two main stages (Fig. 2). The first stage of the region proposal network comprising of identifying the object bounding boxes Next, the highest-ranking bounding boxes are identified and used to generate region proposals. The mask generated by the segmentation model had some noise that included non-relevant subbranches of the main artery and noise due to other cardiac structures. We applied post processing step to further improve the segmentation mask. The proposed framework segments the region of interest and identifies the calcium by applying localization to segment different voxel regions. To achieve this, we branched two instances of the architecture, the first runs a segmentation subnetwork for identifying heart and aorta region and the second runs a subnetwork which identifies epicardial fat and thus is integrated to the above segmentation to improve the precision of multiorgan localization. To segment individual arteries, we use the fact that epicardial fat surrounds the arteries and has a particular density range, which allows us to obtain an epicardial fat mask and hence to segment the arteries. Next, we segment the calcium in the heart by first using intensity threshold to filter calcium in the non-contrast CT and then removing noise as well as non-useful calcium in the bone and outside the heart. Applying the coronary artery masks to the segmented calcium then gives us calcium segments in each artery. The AS for each artery is then computed as the sum of ASs of calcium group in that artery across all slices.

#### 2.7.1 Cardiac and aorta segmentation

**Fig. 3:**
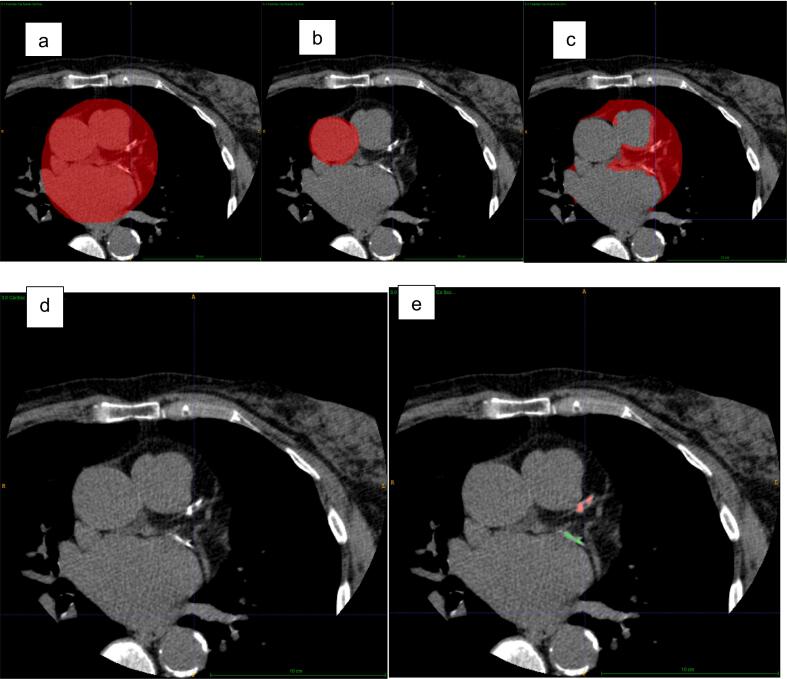
Examples of gated CT study showing Segmentation; a: Cardiac Segmentation; b: Aorta Segmentation; c: Epicardial Fat Segmentation; and d: Showing Coronary Artery Calcification; e: Model detected Coronary Artery Calcification and different colour in coronary artery territories. Red colour and green colour showing LAD and LCx respectively.

We performed cardiac and aorta segmentation. Masks generated by experienced cardiologists were used to train the model (80 cases with 40–50 slices in each mask were used to train the model). Aorta segmentation was performed on the axial slices. Axially the shape of the aorta changes from circular to elliptical to semi-elliptical before it disappears. We use these shape changes in the prediction for the mask, which yields a segmentation (Mean Dice score (0.91). (Similarity between a predicted segmentation mask and the ground truth segmentation mask done by operator.0 indicating no overlap, to 1, indicating perfect overlap). Figs. 3a and b show sample heart and aorta segments, respectively.

#### 2.7.2 Coronary artery region segmentation using epicardial fat

The coronary arteries lie in and are surrounded by the epicardial fat on the epicardial surface; therefore, we locate them in each region with the help of epicardial fat mask. Epicardial fat has a fixed range of density, which allows us to derive a mask for it from the heart mask. Fig. 3c shows a sample epicardial fat segment. Coronary artery regions are then located using dilation and erosion on the derived epicardial fat mask. Finally, heart region splitting is used to locate individual coronary artery regions.

#### 2.7.3 Heart region splitting and coronary artery segmentation

As CAC quantification needs to be specific to the type of artery, we divide the heart into regions containing the corresponding artery. Iterating from the top, the first slice containing elliptical aorta is chosen as a reference and a slice a few millimetres below is used as localization for splitting the heart into various regions. In the remaining slices, we segment the aorta but retain the artery regions, which divides the slices into aorta, right coronary artery (RCA), left anterior descending (LAD), and left circumflex (LCX) regions. (Table 1).

**Table 1:** Coefficients for density ranges used to compute Agatston scores. HU: Hounsfield Units.

**Density range**	**Coefficient**
130–200 HU	1
200–300 HU	2
300–400 HU	3
400 + HU	4

#### 2.7.4 Coronary artery and aorta calcium segmentation

For CAC scoring in coronary arteries and ascending aorta, we need to segment the relevant calcium. To filter calcium, we use an intensity threshold (intensity >130 Hounsfield Units (HU) in non-contrast CT. This filtered calcium also includes non-relevant calcium (noise, artifacts, and bones). We eliminate most of the noise using 2D erosion and dilation and the rest using a volume and maximum intensity threshold. Next, to eliminate bone calcium, we apply 3D dilation only on calcium groups with volume greater than 10,000 voxels (3000 mm^3^) to merge the tiny bone calcium groups into larger ones and eliminate them together. In the above process, some parts of relevant calcium groups may have been removed; we use connected components technique to recover those parts. Finally, we apply the heart mask to segmented calcium to eliminate extra cardiac calcium groups. Fig. 3d, and 3e shows a sample calcium segment in the coronary arteries. On this segmented cardiac calcium, we apply aorta and individual coronary artery (RCA, LAD, and LCX) masks to obtain calcium segment in each artery. Once again, we use connected components technique to recover relevant calcium that may have been lost.

#### 2.7.5 Computing Agatston score (AS)

The AS for each of the aorta and coronary arteries is obtained by summing the ASs for the calcium groups in the artery in each slice and summing these scores over all slices. The AS for an individual calcium group (lesion) is computed by multiplying the lesion area with the corresponding coefficient in Table 1, which assigns a coefficient to the intensity range in which the point of maximum intensity in a lesion lies.

Above-described process identifies the CAC and exclude all non-coronary cardiac calcifications. All the CAC were then cumulatively added, and risk categories were obtained.

#### 2.7.6 Statistical analysis

Statistical analysis was performed using the Statistical Analysis System (SAS) software, version 9.4 (SAS institute, Cary, NC, USA). To compare ASs between automated model and ground truth, Diagnostic performance metrics (sensitivity, specificity, positive predictive value (PPV), negative predictive value (NPV), and F1) at various CAC score cutoffs were calculated for the automated model. Pearson correlation coefficient was used to analyse the similarity of distributions from the automated model and the ground truth. Confusion matrix is used to show correlation of bucketed scores (0-IV, for ASs of 0, 1–100, 101–400, 401–1000, and >1000 respectively).

## 3 Results

1442 patients were tested for the validation of the algorithm (mean age 60+/-10 year, 61% males). Automatic cardiac and aortic segmentation model worked well (Mean Dice score: 0.91). Cohen’s kappa coefficient between the reference actual and the interclass computed predictive categories on the test set is 0.72 (95% CI: 0.61–0.83).

**Fig. 4:**
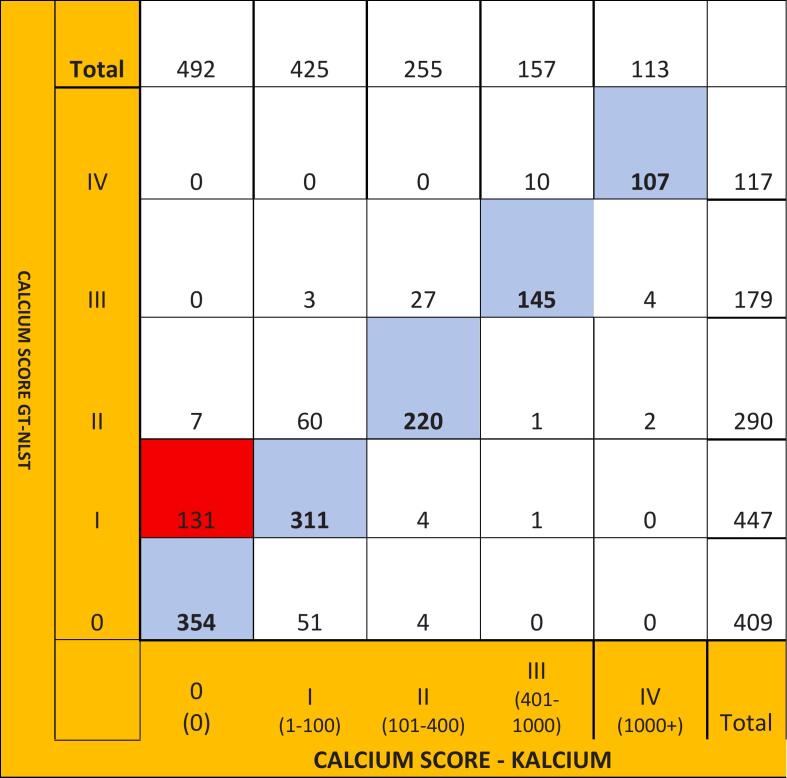
Confusion Matrices showing the Agreement in cardiovascular risk categorization 0: 0, 1: 1–100, II: 101–400, III: 401–1000 and IV: >1000Agatston score) based on Agatston score predicted by the algorithm and model *vs* the reference and Ground Truth.

Diagnostic performance of the model is shown in Table.2. The proposed method correctly classifies the risk group in 78.8% of the cases and classifies the subjects in the same group. F-score (a metric used to evaluate the performance of Machine Leaning Model: 0.7 or higher is considered a good score) is measured as 0.78; 0.71; 0.81; 0.82; 0.92 in calcium score categories 0, I, II, III, IV, respectively. The confusion matrix is shown in Fig. 4. 79% of the predictive scores lie in the same categories, 20% of the predictive scores are one category up or down, and only 17 (1.2%) patients out of 1142 were more than one category off. The risk group is over-estimated in 16.5% of the cases and under-estimated in 8.1%. The main reason for underestimation is a false negative CAC and is especially visible in cases classified as Group 0 automatically while they belong to Group I (131 cases).

**Table 2:** Diagnostic performance of the model.

**Score Category**	**Sensitivity** **(%)**	**Specificity** **(%)**	**Positive Predictive Value (%)**	**Negative Predictive Value (%)**	**F score**
**0 (0)**	0.865	0.866	0.719	0.942	0.789
**I (1**–**100)**	0.695	0.885	0.731	0.866	0.711
**II (100**–**400)**	0.758	0.969	0.862	0.941	0.819
**III (401**–**1000)**	0.810	0.990	0.923	0.973	0.820
**IV (>1000)**	0.914	0.995	0.946	0.992	0.924

## 4 Discussion

CAC scoring is routinely done for CVD risk categorization utilizing non contrast ECG-Gated CT examinations. However, CAC could potentially be assessed using non contrast chest CT studies performed for other indications, such as lung cancer screening and to detect other lung pathologies. We have described fully automated deep learning based coronary calcium scoring solution for Non-Contrast Chest CT and validated it on 1442 patients. We have used Mask R-CNN for multiorgan segmentation. The novelty of the proposed solution lies in post-processing of aortic segmentation to exclude non coronary calcium. Another novelty is noise reduction by epicardial fat segmentation to reduce noise and increase accuracy of the coronary artery calcium detection; 79% of patients were correctly assigned to the right Agatston risk category in our study.

For assessment of CVD risk, it is relevant to distinguish between presence and absence of CAC [25]. When compared with Agatston scoring as ground truth for the presence/absence of coronary artery calcifications, our deep learning model achieved a sensitivity of 90% and a specificity of 94%. Therefore, our model is clinically relevant.

Coronary artery disease is a major cause of mortality globally, and atherosclerotic changes in the coronary artery constitute the main pathophysiology of CAD [[4], [26]]. CACS reflects an atherosclerotic burden and independently predict coronary events [27]. Conventionally, CACS is calculated on ECG-gated non-enhanced calcium score CT with predefined parameters [28]. However, it is possible to calculate CACS based on chest CT images. In 2016, the Society of Cardiovascular Computed Tomography (SCCT) and the Society of Thoracic Radiology (STR) recommended coronary artery calcium scoring based on non-enhanced chest CT images in a jointly published guideline [4]. Many studies have been conducted on CACS acquisition without ECG-gated non-enhanced coronary calcium-scoring CT. Some studies showed a correlation between CACS from non-ECG gated chest CT [[26], [28]], or even non-ECG-gated low-dose chest CT [[24], [29]], and CACS from ECG-gated non-enhanced coronary calcium-scoring CT.

Some other studies have also tried to assess the feasibility and accuracy of automated CAC detection and quantification from non-contrast chest CT. Lee et al. [30] has shown the feasibility of assessment of coronary artery calcium score (CACS) from chest CT with slice thickness of 1.0–1.25 mm as compared to ECG gated cardiac CT with slice thickness of 2.5 mm. They have shown the correlation coefficients of CACS between the coronary calcium-scoring CT with the 2.5 mm and 1.25 mm images were 0.888 and 0.904, respectively.

Gonzalez et al. [15] has described automated Agatston score calculation from non-ECG gated CT chest utilizing CAC segmentation and correctly identified 67.3% of the cases. This study has shown 28% of patients are misclassified in a group one level above or below their correct group and 4.3% of the patients are misclassified with two or more distance groups. They have used automatic heart localization and thresholding for calcium detection on non-ECG- gated chest CT. Our results show improved performance because of less false positive cases and due to novel approach of multi organ segmentation. Same group [17] has shown improved and correct identification of the Agatston score and the risk category in 76.2% of the cases utilizing 3 D deep convolutional neural network like our study.

Sanne et al. [18] has evaluated deep learning (DL) based automatic calcium scoring calculation in multiple Cardiac CT and Chest CT protocols. They have shown that at baseline, the DL algorithm yielded Inter Class Correlations (ICCs) of 0.79–0.97 for CAC across the range of different types of CT examinations. ICCs improved to 0.84–0.99 for CAC and for CT protocol–specific training and to 0.85–0.99C.AC. This study supports the use of protocol specific training and validation, and this was applied in our group. We have recently published data on automatic AI based detection of CAC on ECG- Gated Cardiac CT [31] and this data on Non-ECG- Gated chest CT is comparable.

Chamberlain et al. [19] has shown high performance of AI CNN model (AI-RAD Companion, Siemens Healthineers, USA) to automatically detect coronary artery calcium volume (CACV) from low dose chest CT as compared to results by experienced radiologists with high sensitivity and specificity (sensitivity:0.92; specificity:0.96).

Recent study has used multi-task neural network for performing the segmentation of calcifications on the segment level as the main task and the segmentation of coronary artery segment regions with weak annotations as an auxiliary task. This model shows accuracy of 73.2% to the correct coronary artery segment. The model achieved a sensitivity of 0.732, specificity of 0.978, and a F1-score of 0.717. The segment-level agreement was good with a weighted Cohen’s κ of 0.808 [20].

One other study has shown no significant differences were found in CACS quantification obtained using automatic or manual methods at total and vessel-specific levels [21].

Traditionally, ECG-gated non-contrast CT has been used for the assessment of coronary calcium. Above mentioned studies have shown CAC scores could also be derived from the chest CT performed for other indications, such as lung cancer screening and other indications, thus making it easier and practical to use of AI algorithms in large sets of screening data. Fully automated ML- and DL-based quantification of CAC has been evaluated in large data sets of low-dose, ungated CT scans with high sensitivity and specificity as mentioned earlier. These findings provide support to the use of such a DL method to aid clinicians in CAC scoring across a wide range of non-contrast chest CT images. While AI enables direct computation of the CAC measures from CT images, the Agatston score quantified by conventional methods can also be used as an input into AI models for risk prediction.

CAC scoring of non-gated examinations has been shown to correlate well with scores obtained from ECG gated cardiac CT scans [30]. Ordinal scoring based on a semi-quantitative analysis has correlated well with CAD outcomes. From US perspective, CAC score can potentially be reported from the approximately 15 million annual diagnostics non contrast CT (NCCT) examinations with CVD risk predictions [[25], [32]]. However, since the CAC information is always in the field of view and analysis is simple and quick, reporting CAC on every NCCT examination is feasible. Until recently, there was no specific recommendation for the reporting of CAC on NCCT examinations or for the preferred methods of analysis. There is some data evaluating the extent of the underreporting of CAC from non-gated non-contrast studies. In one study CAC was present in 58% of the CT examinations in 355 patients with known or suspected CAD. Of these, 44% were not reported. Only 1 of 139 patients with left main CAC and 6 of 188 patients with left anterior descending CAC were identified [33]. In a second study, the presence of any CAC was noted by expert reader interpretation in 108 of 201 (53%) NCCT examinations in patients without suspected CAD. However, only 69% of the 108 positive scans were described in the CT report [34].

Similar data is available form UK and >5.5million CT scans were performed in the UK in 2018–19, and the estimate of ∼950 000 thoracic CT scans being performed annually in the UK [35] A significant increase in the number of low-dose chest CT scans in the UK is expected with the expansion of the Targeted Lung Health Checks and screening Program from 2020 [36]. In patients undergoing lung cancer screening, one third of patients are at a high cardiovascular risk but are not identified and not taking statin therapy [37]. The heart is an important component of all imaging involving the chest and a great opportunity to identify high risk individuals and provide primary CVD preventative measures. The identification of calcification in the coronary arteries can provide information on the presence of previously unknown coronary artery disease and trigger an assessment of cardiovascular risk factors or associated symptoms such as chest pain. Automated detection and quantification of CAC has shown significantly higher accuracy as compared to current gold standard of visual reporting of CAC.

Further deep inspection and appraisal of our results revealed that our AI model sometimes segmented small false-positive lesions, mostly representing noise in the vicinity of the coronaries, more often than the baseline, resulting in incorrect CVD risk categorization in the lowest risk categories. Deeper analyses of our false positive results found that the underestimation of the CAC score most frequently occurred in patients with Agatston score ranging from 1 to 100, especially in patients with the CAC category of 1 (F score of 0.71). We have noticed that the coronary artery involvement with smaller or less dense calcium was easily misclassified due to motion artifact, imaging noise, or partial volume effects.

Other studies have also shown Automatic CAC quantification on non-gated chest CT is particularly cumbersome because of high noise, low resolution, and motion artifacts [[38], [39]]. Some other segmentation studies such as pseudo-labelling with dual consistency regularization based on a high capability of uncertainty awareness [40] and Unsupervised Mask-guided annotated CT Image Synthesis with Minimum Manual Segmentations [41] have shown accurate segmentation of the CT images [41].. These studies have shown that semi-supervised learning can learn from limited labelled data and a large amount of unlabelled data, which has shown great potential. Also, compared with the segmentation mask guided synthesis, unsupervised mask-guided synthesis could provide high-quality synthetic images with significantly higher fidelity, variety, and utility in clinical practice. Automated segmentation of the multiple organs and tumours from 3D medical images using novel 3D large-kernel (LK) attention module has shown accurate multi-organ segmentation and tumour segmentation [42]. This module also decomposes the Large Kernel (LK) convolution to optimize the computational cost and can be easily incorporated into convolutional neuronal networks (CNNs) such as fully convolutional network (U-Net). A recent study has shown utility of inter-cascade generative adversarial network (JAS-GAN), namely JAS-GAN, to segment the unbalanced atrial targets from Late Gadolinium Enhancement Cardiac Magnetic Resonance (LGE CMR) images automatically and accurately in an end-to-end way [43]. JAS-GAN investigates an adaptive attention cascade to automatically correlate the segmentation tasks of the unbalanced atrial targets and an adversarial regularization is applied to the segmentation tasks of the unbalanced atrial targets for making a consistent optimization. Compared with the state-of-the-art methods, JAS-GAN has shown better average Dice Similarity Coefficient (DSC) values. Unlike other organs cardiac substructures are proximate to each other and have indiscernible boundaries (i.e., homogeneous intensity values), making it difficult for the segmentation network focus on the boundaries between the substructures. Study has shown promise with shape-aware attention module, that exploits distance regression, which can guide the model to focus on the edges between substructures so that it can outperform the conventional contour-based attention method with good results. Onan [[44], [45], [46]] has shown promising results in text classification and segmentation based on advanced computing and deep learning techniques with efficient and accurate results. However, we have also used multimodality, Multi segmentation model to segment the heart, aorta, coronary artery location, and epicardial fat to reduce the false positives and improve the efficacy. We have also used a segmentation architecture based on Mask R-CNN with high dice score and widely used by other investigators in cardiac segmentation on CT scans.

Other studies have shown clinical application and detection of Tuberculosis (TB) from chest X-ray and Lung cavitation for CT Chest by application of automated automatic recognition method based on hybrid resampling and multi-feature fusion strategies [47]. Ayaz et al. [48] has shown a fully automatic computer-aided diagnosis system can reduce the need of trained personnel for early diagnosis of TB using chest X-ray images. They have proposed a novel TB detection technique that combines hand-crafted features with deep features (convolutional neural network-based) through Ensemble Learning.

There are several limitations with this model. Although the current algorithm for segmentation was trained and tested on multicentre NLST data, the validation was performed on random data originating from few institutes. Future validation on a multi-centre dataset should prove the generalizability of this algorithm. Secondly, limitations associated with a retrospective study design are present. Thirdly, deep learning model for segmentation was trained on 80 subjects and inherent to some bias and precision. Fourthly, only one reader was used as ground truth, with some limitations in terms of consistency. However, our data suggests high similarity to the ground truth.

### 4.1 Scope for future work

In the future study, a more comprehensive segmentation model should be developed for the consideration of non-coronary artery cardiac calcification and aortic calcification. This will reduce falls positive results. Pre-processing of images needs further refinement to avoid noise to be picked as small calcium and improve the performance of the model for detection of low levels of coronary calcium. A further multi-centre and multi-scanner study should be carried out to verify the proposed automated framework. Prospective, multicentric, and prognostic studies are required to validate the algorithm and its clinical impact on risk stratification and prevention of cardiac events.

### 4.2 Conclusion and practical clinical application

In conclusion, we have demonstrated our deep learning based automated calcium detection and quantification on chest CT show high correlation with manual calcium detection and Agatston scored from dedicated software. Automated analysis could increase the workflow efficacy and help deal with the increasing number of CT requests to assist in the increased workforce of radiologists. Automation could also potentially improve the efficacy and reporting of incidental CAC during non-contrast CT scan. This would be especially important in screening situations and particularly in low- and middle-income countries where there is shortage of trained CT cardiologists and radiologists. This will also identify the patients with subclinical CAD and high risk for future cardiac events from non-ECG gated CT chest. Aggressive primary prevention measures such as lipid lowering therapies could be applied to reduce cardiac events and burden of heart disease. Recent advances in artificial intelligence (AI) and machine learning algorithms, using automation to analyse CT scans and consistent interpretations has become reality. Such algorithms, if implemented appropriately, could assist physicians and lead to early diagnosis and improved patient care.

## Availability of data and materials

The data that support the findings of this study are not openly available due to reasons of sensitivity and are available from the corresponding author upon reasonable request. Data are located in controlled access data storage at KARDIOLABS.AI, Jacksonville, USA.

## CRediT authorship contribution statement

**Sudhir Rathore:** Writing – review & editing, Writing – original draft, Validation, Supervision, Software, Resources, Methodology, Formal analysis, Data curation, Conceptualization. **Ashish Gautam:** Data curation. **Prashant Raghav:** Formal analysis, Data curation. **Vijay Subramaniam:** Data curation. **Vikash Gupta:** Data curation. **Maanya Rathore:** Data curation. **Ananmay Rathore:** Data curation. **Samir Rathore:** Data curation, Conceptualization. **Srikanth Iyengar:** Writing – review & editing, Conceptualization.

## Author contribution

All authors contributed to: (1) substantial contributions to conception and design, or acquisition of data, or analysis and interpretation of data, (2) drafting the article or revising it critically for important intellectual content, and (3) final approval of the version to be published.

## Funding

Prashant Raghav and Samir Rathore are employees of the Kardiolabs AI and hold stock options in the company and do not have any role in the design and analysis of the study. No other Funding sources.

## Declaration of competing interest

The authors declare that they have no known competing financial interests or personal relationships that could have appeared to influence the work reported in this paper.
